# Potent Antitumor Activity Generated by a Novel Tumor Specific Cytotoxic T Cell

**DOI:** 10.1371/journal.pone.0066659

**Published:** 2013-06-18

**Authors:** Zheng Wang, Pei Li, Qinhong Xu, Jun Xu, Xuqi Li, Xufeng Zhang, Qingyong Ma, Zheng Wu

**Affiliations:** Department of Hepatobiliary Surgery, First Affiliated Hospital of Medical College, Xi'an Jiaotong University, Xi'an, Shaanxi, China; University of Chicago, United States of America

## Abstract

Hepatocellular carcinoma is one of the most common malignant neoplasms in the world and is the main cause of death in patients with liver cirrhosis. Surgical intervention is not suitable for majority of hepatocellular carcinoma. Investigation of the effective targeting to the tumor cells is essential for both primary tumors and metastases. Tumor specific cytotoxic T lymphocytes (CTL) have been considered to be the attractive vehicles for delivering therapeutic agents toward various tumor diseases. This study was to explore the distribution pattern of CTL carrying the lentiviral vectors with the characteristic of adenoviral E1 gene under the control of the cell activation-dependent CD40 ligand promoter (Lenti/hCD40L/E1AB). Following transduction with adenoviral vectors containing chimeric type 5 and type 35 fiber proteins (Ad5/35-TRAIL), these CTLs produced infectious virus when exposed to HepG2 cells. We assessed the therapeutic ability of CTLs using MTT, Western blot and colony formation assay. The novel CTL harboring Lenti/hCD40L/E1AB and Ad5/35-TRAIL caused proliferation inhibition and significant apoptosis in hepatocellular carcinoma cell lines. Thus, the novel CTL may be useful for the development of gene therapy approaches to hepatocellular carcinoma.

## Introduction

Cancer is the leading cause of death in economically developed countries and the second leading cause of death in developing countries[1]. Hepatocellular carcinoma (HCC) is a common, highly invasive malignant tumor associated with a high mortality rate. It is the third leading cause of cancer deaths worldwide [2], and with the highest incidence rates reported in East Asia [3], [4]. Recurrence, metastasis and the development of new primary tumors are the most common causes of mortality among patients with HCC [5]. The mainstay of therapy is surgical resection, however, only 10–20% of patients being suitable for surgical treatment [6]. Other therapy such as radiotherapy and chemotherapy could not bring the satisfactory effect on the HCC patients. How to deal with the other non-surgical patients is a confused problem. Recently, it is reported that T-cell therapy has the potential to eradicate malignant HCC disease.

Tumor antigen–specific cytotoxic T lymphocytes (CTLs) have been shown in a number of preclinical and clinical models to be highly effective at infiltrating tumor sites in a multiplicity of organs [7], [8]. However, while CTLs may eradicate some types of experimental and natural tumors, it has become evident that many malignancies exhibit a range of immune evasion mechanisms that diminish the effectiveness of attack [9], [10]. Therefore, it is necessary to combine the immunotherapy with virus therapy on the treatment of HCC.

CD40 and CD40 ligand (CD40L) are members of the TNF family, and their interaction provides a potent signal for DC activation. CD40L expression is tightly regulated, being transiently expressed on the surface of activated CD4^+^ T cells for less than 24 hours [11]. The expression of CD40 can induce maturation of CD40^+^ dendritic cell and B lymphocytes. The CD40L promoter is tightly regulated by the AT hook transcription factor AKNA, which is expressed only transiently following antigen-mediated T-cell activation [12], [13]. We reasoned that if this promoter were used to drive the adenoviral E1 gene, expression would occur only after the T cell encountered its target.

In the development of gene therapy, Ad5 vectors transduce predominantly hepatocytes after intravenous injection, and because tumor cells do not often express the Ad5 receptor (coxsackievirus–adenovirus receptor, or CAR), these vectors are unsuitable for tumor targeting therapy. Because commonly used species C Ad serotype 5-based vectors do not efficiently transduce HCC, the chimeric Ad5 vectors carry fibers from species B Ad serotype 35 (Ad5/35) [14]. These vectors infect cells through CD46, a protein whose expression is upregulated in majority undifferentiated cells, including HCC [14], [15]. We used a chimeric adenoviral vector in which the fiber protein of Ad5 is substituted by the fiber of Ad35. This Ad5/35 vector is CAR independent and transduces human T cells [16]. TRAIL (Tumor Necrosis Factor Related Apoptosis Inducing Ligand) is a highly promising anti-cancer agent with pronounced pro-apoptotic activity towards various malignant cell types, including lung cancer. Importantly, TRAIL essentially lacks activity towards normal cells [17]. TRAIL, via the extrinsic apoptotic pathway, engages its receptors, recruits caspase 8, which is then cleaved to its active form. Activated caspase 8 then cleaves the BH3-only molecule, Bid, which then interacts with mitochondrial anti- and proapoptotic molecules.

In this study, we constructed a novel CTL harboring LV-CD40Lpr and Ad5/35-TRAIL which caused proliferation inhibition and significant apoptosis in hepatocellular carcinoma cell lines. We wonder if this kind of CTL could be applied in the HCC treatment.

## Materials and Methods

### Ethical statement

The study protocol and consent forms conform to the Declaration of Helsinki and were approved by the Ethical Review Board (ERB) Committee (The First Affiliated Hospital of Medical College, Xi'an Jiaotong University, China) and informed consent was obtained from all subjects. All the participants provided their written informed consent to participate in this study under the inspection of ERB committee.

### Cell culture

The human embryonic kidney cell line (HEK293), HEK293T, human hepatocellular carcinoma cell line (HepG2) were obtained from the American Type Culture Collection (Rockville, MD), and cultured in Dulbecco's modified Eagles medium (DMEM) supplemented with 10% FBS and 100 IU/ml penicillin/streptomycin in a 37°C humidified incubator with 5% CO_2_.

### Production of lentiviral vectors

The CD40L promoter was cloned from human genome using the specific primers (upstream: 5′- CCCAAGCTTAAGAAAGCAGGTGCTAACTATATAG-3′ and downstream: 5′- CGGGATCCGCTGTGTTAAAGTTGAAATGGTATC-3′) and subcloned into the lentiviral shuttle vector pLenti, named pLenti/hCD40L. The E1 gene (Ela, Elb) was polymerase chain reaction amplified and subcloned into the downstream of pLenti/hCD40L vector, pLenti/hCD40L/E1AB. Lentiviral particles were produced by performing transient co-transfection involving a three-plasmid expression system in HEK293T cells according to user's manual, and concentrated through ultracentrifugation.

### Production of adenoviral vectors

According to the previously reports, we constructed the Ad5-GFP and Ad5/35-TRAIL vectors [18]. Ad5/35-GFP is an E1/E3-deleted adenoviral vector targeted to CD46 by the adenoviral serotype 35 fiber. To construct the Ad5/35-GFP vector, we inserted the green fluorescent protein (GFP) and IRES-TRAIL gene into pAd35CMV [19] downstream of the cytomegalovirus (CMV) promoter. This plasmid was then cotransfected together with pAd35Helper into HEK293 cells to generate recombinant virus (Ad5/35-TRAIL) [19]. Adenoviral vectors were purified by ultracentrifugation in CsCl gradients. The ratio of viral particles to plaque-forming units was for all vectors 15∶1. Each viral stock produced was tested for endotoxin contamination by the Limulus amebocyte lysate assay (Associates of Cape Cod, East Falmouth, MA).

### Lymphocytes isolation and flow cytometry identification

Lymphocytes was isolated from human blood using the human lymphocyte separation medium, then transferred to a 25 cm^2^ plate (Costar), precoated with RetroNectin (2 µg/L; TaKaRa, Japan) and anti-CD3 antibody (50 ng/ml; TaKaRa, Japan) at 1×10^6^ cells per well, and incubated for 48 hours for optimal activation before transduction. The stimulated CTL lines were resuspended at 1×10^6^ cells/mL in complete medium supplemented with IL-2 (300 U/ml), INF-γ (400 U/ml), phytohaemagg lutinin (10 U/ml), rhIL-4 (100 U/ml), GM-CSF (10 U/ml) and rhIL-2 (100 IU/mL), and then incubated for 36 hours at 37°C and 5% CO_2_. CD3-FITC, CD4-PE, CD8-PE, CD56-PE, CD226-FITC, CD11-PE and CD305-FITC monoclonal antibodies (MAbs) with the appropriate fluorescein isothiocyanate (FITC)– or R-phycoerythrin (PE)–conjugated were purchased from BD Bioscience (Mountain View, CA). Cells were washed and stained with the appropriate antibodies for 20 minutes at 4°C in the dark in phosphate-buffered saline (PBS) supplemented with 0.1% bovine serum albumin (BSA). Control cells were unstained. After incubation, the cells were washed twice and resuspended in PBS.

### Transduction of CTLs

The CTL lines were resuspended at 1×10^6^ cells/mL in complete medium, and then incubated with different volumes (0.1, 1, 10, 100 µL) of freshly generated Lenti/hCD40L/E1AB for 36 hours at 37°C and 5% CO_2_. To increase the efficiency of transduction, 2 to 3 rounds of transduction were performed. The transduced CTLs were subsequently maintained in Yssel medium (Gemini Biological Products, Calabasas, CA) supplemented with 10% serum. Except where stated, cells were transduced at 37°C with 10^3^ vp per cell in Opti-MEM medium. At 6 hours after transduction, the cells were washed in 4 mL PBS and resuspended in fresh medium supplemented with 10% FCS.

### Western blot

CTLs were transduced with the Lenti/hCD40L/E1AB as described above, and then added into HepG2 cells for 48 h; the total cells were lysed using cold radioimmunoprecipitation assay buffer [20 mmol/L Tris-HCl (pH 8.0), 100 mmol/L NaCl, 10% glycerol, 1% NP40, 0.5% sodium deoxycholate]. After sonication, the lysates were centrifuged at 10,000 g for 10 minutes. Protein concentrations were determined by commercial assay (Bio-Rad, Hercules, CA). Proteins were separated on a 12% SDS-PAGE under denaturing conditions and transferred to a supported PVDF membrane (BioRad, Hercules, CA). The membrane was blocked for 1 h at room temperature in TBS-T [50 mmol/L Tris-HCl (pH 7.5), 150 mmol/L NaCl, 0.1% Tween 20] buffer containing 5% nonfat milk. Membranes were then incubated overnight at 4°C or 1 h at room temperature with the respective primary antibodies: Adenovirus E1A (1∶500) and GAPDH (1∶500). Anti-mouse secondary antibody conjugated to horseradish peroxidase (Santa Cruz Biotechnology) was used to visualize the stained bands with an enhanced chemiluminescence visualization kit (GE Healthcare Life Sciences). The signal was detected by incubation with chemiluminescent substrate and developed on FX7 (Vilber, France).

### MTT Assay

The cytotoxic activity of Lenti/hCD40L/E1AB and Ad5/35-TRAIL on the CTLs was determined by an MTT assay. Briefly, cells were seeded in 96-well tissue culture plates at a density of 5×10^3^ cells/well and then treated with Lenti/hCD40L/E1AB and Ad5/35-TRAIL at the suitable MOIs in the growth medium for 24, 48 and 72 hours. The following day, the medium was removed, and 100 µL of fresh medium containing 0.5 mg/mL MTT (Roche; Manheim, Germany) was added to each well. The cells were incubated at 37°C in humidified 5% CO_2_ atmosphere for 4 hours, followed by the addition of 150 µL of solubilization solution (0.01 mol/L HCl in 100 g/L sodium dodecyl [SDS]) to each well, and the incubation of cells for a further 10 minutes at 37°C with gentle shaking. The optical density of the plates was measured using the spectrophotometrical absorbance at 570 nm in the Microplate Reader Model 550 (Bio-Rad; CA).

### Effect of new CTLs on HUVEC tube formation

To evaluate anti-angiogenic effects mediated by new CTLs, we used the new CTLs in subsequent experiments. To obtain the new CTLs, CTLs cells were seeded on 100- mm dishes containing medium with 10% FBS. After 24 hours, the medium was added into Lenti/hCD40L/E1AB and Ad5/35-TRAIL with the suitable MOIs, then, the cells was collected after 24 hours. Then, HUVEC were cultured in M199 medium in each treatment and their tube formation were evaluated in the microscope.

### Colony formation in soft agar

To assess anchorage-independent growth, soft agar clonogenic assays were done. Each well of a 6-well plate contained 2 mL of 0.5% (w/v) Noble agar (Difco) in DMEM with 10% NBCS. HepG2 cells and the indicated CTLs were mixed equally, and 3×10^3^ cells in 2 mL of 0.375% (w/v) Noble agar in 10% NBCS DMEM were added above the polymerized base solution. All solutions were kept at 40°C before pouring to prevent premature agar polymerization and to ensure cell survival. Plates were incubated (37°C, 5% CO_2_) under standard conditions for 10 days before colony number and diameter were quantified microscopically.

### Statistical analysis

All data are presented as means ±SD of multiple replicate experiments. Intergroup differences were analyzed using paired t testing. P values of 0.05 and below were taken as significant.

## Results

### Isolation and identification of lymphocytes by flow cytometry

**Figure 1:**
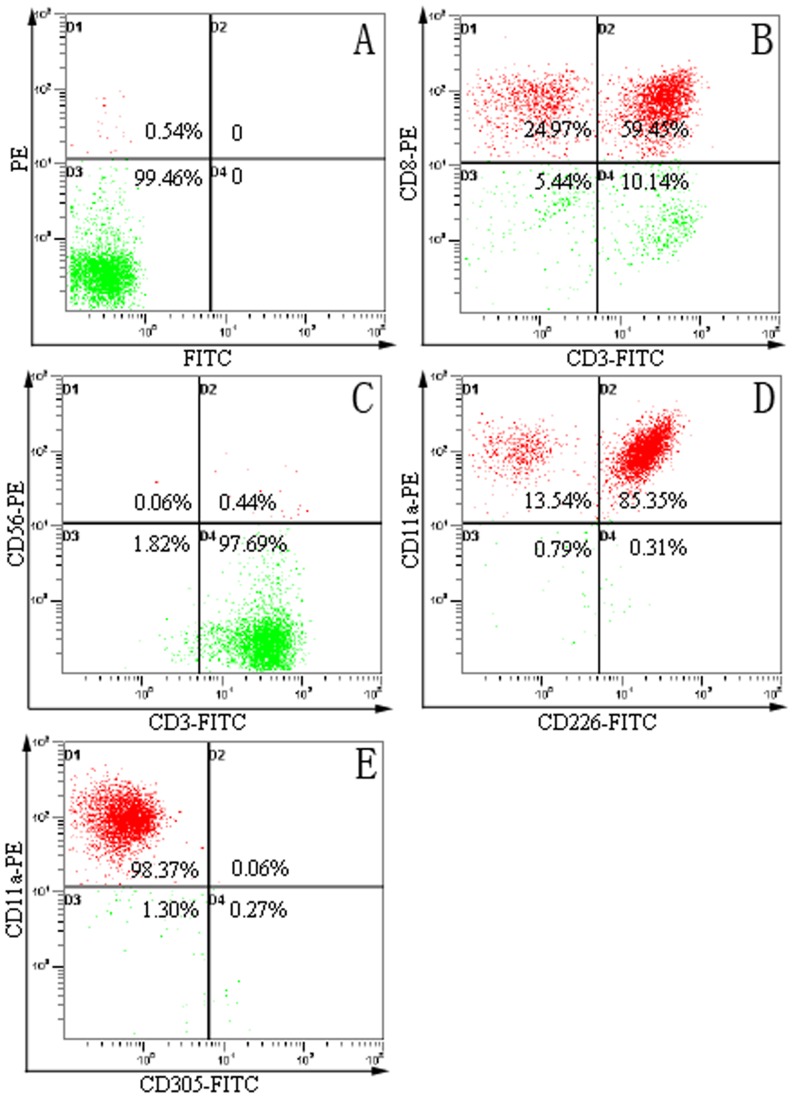
CTLs phenotype was identified by flow cytometry. We used the flow cytometry to detected the phenotype of CTLs. (A) control CTLs without labeled; (B) CTL were stained with monoclonal antibodies specific for CD3 (FITC labeled) and CD8 (PE labeled); (C) CTL were stained with monoclonal antibodies specific for CD3 (FITC labeled) and CD56 (PE labeled); (D) CTL were stained with monoclonal antibodies specific for CD226 (FITC labeled) and CD11a (PE labeled); (E) CTL were stained with monoclonal antibodies specific for CD305 (FITC labeled) and CD11a (PE labeled).

In order to identify the CTL cells, we examined the expression of CD3, CD8, CD11a, CD56, CD226 and CD305 expression on the cell surface of CTL cells. After isolation and incubation, the cells were stained with antibodies to T cell markers (CD3-FITC and CD8-PE) and a mixture of FITC- or PE-labeled antibodies to CD56, CD226, CD11a and CD305 (CD56-PE, CD226-FITC, CD11a-PE and CD305-FITC). CD3, CD11a, CD56 and CD226 were expressed in the CTL cells surface, and CD8 and CD305 were negative (Figure 1). These resultsindicated the isolated cells have the characteristic of CTLs.

### Preparation of lentivirus transduced adenoviruses producer CTL cells

**Figure 2:**
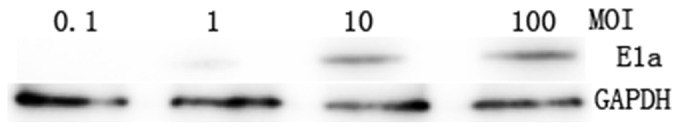
E1a protein is detected in CTL after infected with different MOI of adenoviruses. Western blot analysis: The CTLs were infected with 0.1, 1, 10 and 100 MOIs of adenoviruses, then lysed, and the proteins were separated on a gel. E1a monoclonal antibody was used to detect the target protein.

The schema for generating CTLs capable of producing adenovectors following the lentiviruses (Lenti/hCD40L/E1AB) was described in material and methods. We first transduced the CTLs with a Lenti/hCD40L/E1AB containing the adenovirus E1 gene driven by the CD40L promoter (named CD40L-CTLs). To determine the suitable MOIs of CTL transduction by the Lenti/hCD40L/E1AB, the cell lines were analyzed by western blot using a monoclonal antibody specific to adenoviral E1a. After infected with the Lenti/hCD40L/E1AB at different MOIs, the CD40L-CTLs expressed the E1a adenovirus protein within 48 hours was confirmed by western blot analysis of cell lysates (Figure 2).

### Function of adenovirus -transduced CD40L-CTLs

**Figure 3:**
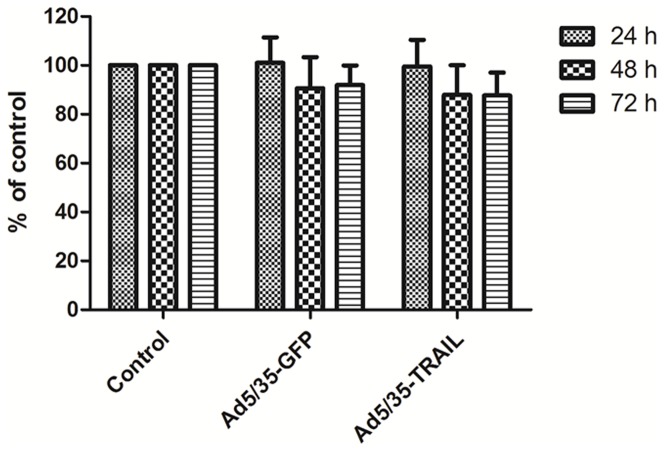
Adenovirus-transduced CTL function. The graphs illustrate the proliferation rate of nontransduced and transduced CTLs (CD40L-CTLs infected with Ad5/35-GFP or Ad5/35-TRAIL) cultured in minimum media (Yssel pluse 10% FCS) alone by MTT method. The rate of proliferation of CTL lines was not significantly different from the nontransduced or transduced CTL line.

To ensure that adenovirus-transduced CD40L-CTLs continued to amplification, we tested their proliferative activity by MTT assay. CD40L-CTLs were infected with Ad5/35-GFP or Ad5/35-TRAIL in the 96 well plate, and cultured in minimum media (Yssel plus 10% FCS) alone. The cell counts revealed that the proliferation rate was the same for both cultures for 24, 48 and 72 h (Figure 3). The CD40L-CTLs harboring Ad5/35-TRAIL (named Ad-CD40L-CTL) has the similar ability of proliferation compared with controls.

**Figure 4:**
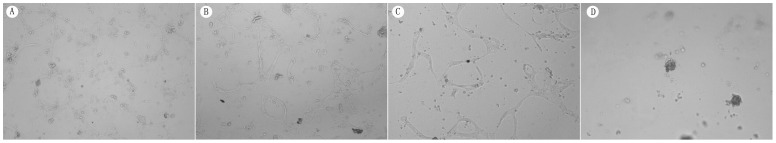
Tube formation detection *in vitro*. *In vitro* angiogenesis assay showed that HUVEC formed vessel like structures (tubes) when plated on ECM wells. Thin and weakly stained tube-like structures could be observed in the CTLs group and CTL infected with Ad5/35-TRAIL adenovirus group. In contrast, Ad-CD40L-CTL caused thinner or only weakly-stained tube-like structures, compared to the control. There was a significant difference in the number of capillary connections. (A) Untreated HUVEC cells; (B) HUVEC cells treated with CTL infected with Ad5/35-TRAIL adenovirus; (C) HUVEC cells treated with CTL; (D) HUVEC cells treated with Ad-CD40L-CTL.

In vitro angiogenesis assay showed that HUVEC formed vessel-like structures (tubes) when plated on ECM wells (Figure 4). HEK293 cells treatment did not inhibit tube formation or network formation. Thin and weakly stained tube-like structures could be observed in the CTLs group and CTL infected with Ad5/35-TRAIL adenovirus group. In contrast, The combination of Ad5/35-TRAIL and CD40L-CTL (named Ad-CD40L-CTL) also obviously inhibited tube formation compared to other groups and their presence was associated only with weakly-stained tube-like structures. There was a significant difference in the number of capillary connections These results suggest that CTL suppresses HUVEC tube formation and that Ad-CD40L-CTL causes further inhibition of this action.

**Figure 5:**
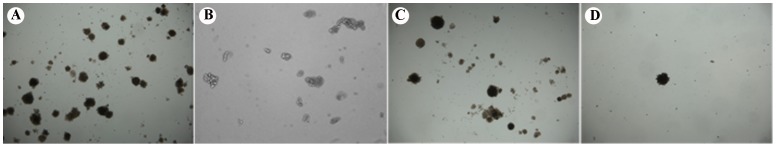
Ad-CD40L-CTL decreases colony formation of HCC cell line HepG2 in soft agar. (A) Untreated HepG2 cells grown in the soft aga; (B) HepG2 cells treated with CTLs infected with Ad5/35-TRAIL adenovirus and grown in the soft agar; (C) HepG2 cells treated with CTLs and grown in the soft agar;(D). HepG2 cells treated with Ad-CD40L-CTL and grown in the soft agar.

We also tested the effects of Ad-CD40L-CTL on colony formation of HepG2 cells in soft agar (Figure 5). Ad-CD40L-CTL treatment strongly inhibited the colony-forming ability of HepG2 cells; both the number and size of colonies were significantly inhibited. We noted that CTL and CTL infected with Ad5/35-TRAIL adenovirus group.had little effect on the proliferation of the HepG2 cells in cell culture. So the effects of Ad-CD40L-CTL were confined to the oncogenic behavior of HepG2 cells, as measured by formation of growth of the cells in soft agar.

## Discussion

Hepatocellular carcinoma is one of the most common malignant neoplasms in the world and is the main cause of death in patients with liver cirrhosis. Surgical treatments including hepatic resection and liver transplantation are considered as the most effective treatment of HCC, however for various reasons, the vast majority of HCC patients are not suitable for surgery.

Inducible gene expression from a non-integrating, non-toxic Ad5/35 vector in HCC has a series of potential applications in cancer gene therapy. We focused our attempts on incorporating the Lenti/hCD40L/E1AB and Ad5/35 expression systems into CTL system. The availability of a chimeric adenoviral vector (Ad5/35) that can infect human T cells and of an activation-dependent promoter (CD40L) controlling a critical early adenoviral replicative gene E1 can made it possible to generate cytotoxic T cells that produce adenoviral vectors when they encounter their target antigen. These antigen-specific T cells retain their specificity and function after exposure to lentiviral vectors encoding the E1 antigen driven by a CD40L promoter and to E1-deleted Ad5/35 vectors encoding eGFP or a gene of TRAIL. Upon antigen-induced activation, the T cells produce infectious virus that can enter proximate target cells. If the adenoviral vector contains a tumor therapy gene TRAIL, the approach can be used to produce a higher level of target cell killing than could be achieved by the CTL alone.

It has been well established that cytotoxic T lymphocytes have the potential to directly kill malignant cells [20], which express and display specific antigenic peptides in the context of specific class I MHC molecules [21], [22]. These antigenic peptides, often referred to as CTL epitopes, are peptides of unique amino acid sequence, usually 9–11 amino acids in length. The tumor-associated antigenic peptide that is being targeted can be used as a peptide-based vaccine to promote the anti-tumor CTL response [23]. However, when the target peptide is derived from non-mutated differentiation antigens as is often the case (e.g. melanosomal proteins), it can be insufficient to engender robust and sustained anti-tumor CTL responses [24], [25]. This is a result of immune tolerance mechanisms that generally suppress or eliminate high avidity auto-reactive T cells [26]. As a result of these mechanisms, the vast majority of tumor-specific CTL, specifically those that recognize non-mutated tumor-associated antigens, are eliminated in the thymus and in the periphery. What remains is a low frequency of tumor-specific CTL, and/or CTL that bear low avidity T cell receptors for the cognate tumor antigen [27], [28].

The stimulation of the CTLs transduced with both E1 lentivirus and E1-deficient Ad5/35 adenoviral vectors could produce substantial quantities of infectious adenoviral vector when these cells were cultivated with CTL target cells. Transduction of target cells was substantially lower when cultivation occurred with CTL targets, consistent with a lower degree of T-cell activation through an CTL-restricted receptor pathway. The adenoviral vector produced was capable of infecting a high proportion of CTL-positive target cells. If the vector encoded a potentially oncolytic gene such as TRAIL, then it was possible to subsequently kill the target cells. The substitution of Ad-CD40L-CTL was capable of producing substantially greater tumor cell death than the use of cytotoxic T cells alone.

In summary, we present the Ad5/35 vector and Lenti/hCD40L/E1AB expression systems that allow for tightly regulated CTLs expression in human HCC. The function and specificity of these vector producer T cells appears unimpaired, and incorporation of an vector encoding a potentially oncolytic gene, such as TRAIL, can increase target killing compared with CTLs alone. It will be of interest to discover whether targeted delivery of adenoviral vectors by cytotoxic T cells will have wider applicability in cancer therapy.
